# 760 Wholistic View of Autografting Patients by %TBSA Burned: Medical Chart Abstraction Integrated with Administrative Claims

**DOI:** 10.1093/jbcr/irac012.313

**Published:** 2022-03-23

**Authors:** Helen D Hahn, Tzy-Chyi Yu, Chia-Chen Teng, Hiangkiat Tan

**Affiliations:** Mallinckrodt Pharmaceuticals, Hampton, New Jersey; Mallinckrodt Pharmaceuticals, Hampton, New Jersey; HealthCore, Inc, Wilmington, Delaware; HealthCore, Inc, SAN DIEGO, California

## Abstract

**Introduction:**

The current literature of severe burns with autografting is scarce or limited to single data source. This study provides a wholistic view of the clinical and economic characteristics of the inpatient treatment of patients with burns using integrated medical chart abstraction with administrative claims.

**Methods:**

Patients with thermal burns undergoing inpatient autografting between July 1, 2010 and November 30, 2019 were identified from large national health plans, representing over 50 million members in the US. The first observed hospitalization with autografting was regarded as the index event. Two hundred hospital patient medical records were abstracted for the clinical characteristics, using a standardized abstraction form, which were integrated with administrative data for economic characteristics. Those with large % TBSA were oversampled. Patients were stratified into three cohorts by %TBSA burned. A Bonferroni correction of alpha 0.017 was performed for post-hoc pairwise tests.

**Results:**

Of 200 patients, 90 were categorized as low TBSA (< 10%), 75 moderate TBSA (10 – 24%), and 35 high TBSA (25+%). Overall, the high %TBSA cohort appeared to be younger, requiring more intensive care, and thereby incurring higher costs of care. The extent of some undocumented critical fields in the medical records was significant, for example (%undocumented): donor size (80%), BMI (31%), mesh ratio (30%), etc.

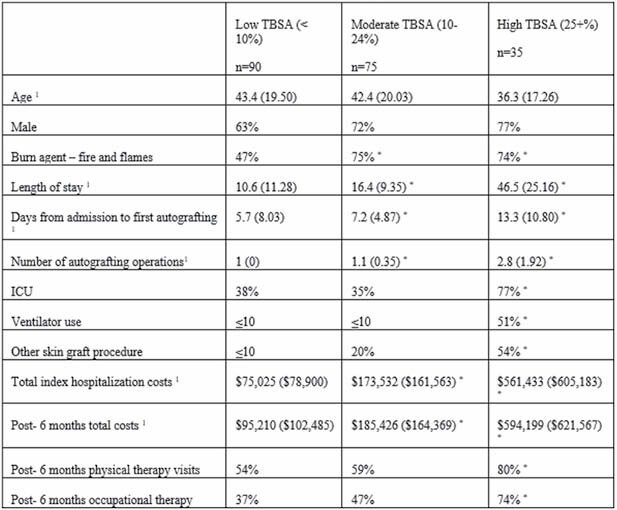

^1^ mean (SD)

^*^ compared with low TBSA, p< 0.017

**Conclusions:**

This study not only confirmed some conventional understanding of various TBSA cohorts, but also quantified their differences/similarities using multiple data sources. There was considerable incompleteness in many critical fields in the medical records, which limits the ability to generate broader insights. Despite the wide regard of medical records as a “gold standard” for outcomes analyses, future research should be aware of these limitations.

